# Efficacy, Safety, and Mechanism of the Qi-Lian-Xiao-Pi Prescription (WW-1) for Chronic Atrophic Gastritis After Helicobacter Pylori Eradication: Protocol for a Multicenter, Randomized, Double-Blind, Placebo-Controlled Trial

**DOI:** 10.2196/90965

**Published:** 2026-07-08

**Authors:** Wei Bai, Guodong Huang, Xianjun Rao, Hao Li, Tingting Zhou, Yang Yang, Wei Wei

**Affiliations:** 1 Department of Gastroenterology Wangjing Hospital of China Academy of Chinese Medical Sciences Beijing, Beijing China; 2 Graduate School Beijing University of Chinese Medicine Beijing, Beijing China

**Keywords:** chronic atrophic gastritis, Qi-Lian-Xiao-Pi prescription, randomized controlled trial, traditional Chinese medicine, WW-1

## Abstract

**Background:**

Chronic atrophic gastritis (CAG) is widely recognized as one of the precancerous lesions of gastric cancer. *Helicobacter pylori* is one of the important risk factors for CAG and gastric cancer. However, a large proportion of patients with CAG cannot avoid developing gastric cancer even after eradicating *H pylori*. It is necessary to find a safe and effective treatment to suppress this “inflammation-cancer” progression. The Qi-Lian-Xiao-Pi prescription (WW-1), a traditional Chinese medicine (TCM), has been reported to be effective in the treatment of CAG. However, the evidence is subject to methodological limitations.

**Objective:**

This study aimed to evaluate the efficacy, safety, and mechanism of the WW-1 in patients with CAG following successful *H pylori* eradication.

**Methods:**

This study is a rigorous parallel-arm, randomized, placebo-controlled, multicenter, double-blinded trial. A total of 110 eligible participants with a confirmed diagnosis of CAG after *H pylori* eradication are being enrolled and randomly assigned in a 1:1 ratio to either the intervention group (WW-1) or the control group (WW-1 placebo). Key eligibility criteria include confirmed CAG by histopathology, documented successful *H pylori* eradication, and compliance with predefined inclusion and exclusion criteria. The treatment duration is 24 weeks. Blinded histopathological assessments using the Operative Link on Gastritis Assessment and Operative Link on Gastric Intestinal Metaplasia Assessment staging systems will serve as primary outcomes. Secondary outcomes include improvement rates of gastric mucosal gland atrophy and intestinal metaplasia, as well as TCM syndrome scores. Safety will be assessed through monitoring vital signs, adverse events, blood, urine, and stool tests, liver and kidney function, and electrocardiography. Additionally, gastric mucosal DNA methylation and metagenomic sequencing of digestive tract microbiota (including saliva, tongue coating, gastric, and intestinal samples) will be analyzed to explore potential mechanisms of WW-1.

**Results:**

The funding began in November 2023. The study was officially initiated on April 20, 2025, with the enrollment of the first participant. The final study results, including efficacy outcomes, safety profiles, and mechanistic insights, are expected to be released in October 2026 after comprehensive data analysis and verification.

**Conclusions:**

This study is designed to determine whether WW-1 can improve CAG by modulating gastric mucosal DNA methylation and the digestive tract microbiota. It represents a prospective clinical trial in TCM that aims to evaluate therapeutic effects on CAG through the regulation of microbiota homeostasis and epigenetic mechanisms. The findings of this study are expected to provide evidence regarding the efficacy and safety of WW-1 and contribute to the development of therapeutic strategies and future drug research for CAG.

**International Registered Report Identifier (IRRID):**

DERR1-10.2196/90965

## Introduction

Chronic atrophic gastritis (CAG) is a common disorder of the digestive system and is widely recognized as a precancerous lesion of gastric cancer (GC) [1]. According to the International Agency for Research on Cancer, in 2022, there were approximately 968,000 new cases of GC worldwide, with over 660,000 deaths, ranking fifth in both incidence and mortality [2]. More than 70% of these cases occur in Asia, with nearly half in Eastern Asia, particularly China. CAG represents a key stage in the progression toward GC, with a prevalence of approximately 25% reported in studies conducted between 2010 and 2020 [3]. Among patients with CAG, the annual risk of developing GC is estimated at 0.1%, with cumulative incidences of 0.6% and 0.8% over 5 and 10 years, respectively [4].

*Helicobacter pylori* infection is the primary cause of CAG, inducing chronic inflammation of the gastric mucosa that leads to glandular loss, mucosal atrophy, and thinning, often accompanied by intestinal and/or pseudopyloric metaplasia [5]. Although *H pylori* eradication reduces GC risk in some populations, evidence indicates that a considerable proportion of patients with CAG remain at risk of progression even after successful eradication [6]. Notably, a large-scale Japanese cohort study (n=148,489) demonstrated that patients with *H pylori*–associated gastritis or gastric ulcer had a 2.0- to 2.4-fold higher risk of GC than those with duodenal ulcer after successful eradication, highlighting that persistent gastric mucosal atrophy is the key determinant of residual cancer risk [7].

The Correa cascade describes the stepwise progression of gastric carcinogenesis from normal mucosa to superficial gastritis, atrophic gastritis, intestinal metaplasia (IM), dysplasia, and ultimately GC [8]. Gastric mucosal atrophy, IM, and dysplasia are well-established independent risk factors for GC and represent critical stages in the inflammation-to-cancer transition. Despite advances in surgery, chemotherapy, radiotherapy, immunotherapy, and targeted therapies, the overall 5-year survival rate for GC remains low, ranging from 20% to 30% [9]. Therefore, effective interventions targeting the early stages of this cascade, particularly CAG, are urgently needed to prevent disease progression.

Current clinical treatments for CAG include acid suppression, *H pylori* eradication, and mucosal protection. However, due to the complex pathophysiology of CAG, these therapies often provide only partial symptomatic relief and limited reversal of histopathological changes. In recent years, traditional Chinese medicine (TCM) has gained increasing attention as a complementary therapeutic approach. Several TCM formulas, such as Xiao-Jian-Zhong-Tang, Sha-Shen-Mai-Dong-Tang, and Jian-Pi-Yi-Qi-Tang, have demonstrated potential benefits in improving CAG in clinical studies [10]. Mechanistically, TCM formulas may modulate multiple pathways, including inflammatory responses, cell proliferation and apoptosis, mucosal metabolism, and tissue repair. Nevertheless, most existing studies are limited by small sample sizes, single-arm designs, or insufficient methodological rigor. High-quality multicenter, randomized, double-blind, placebo-controlled trials remain scarce, despite being the gold standard for evaluating clinical efficacy [11].

Qi-Lian-Xiao-Pi prescription (WW-1), a modified formula derived from Ban-Xia-Xie-Xin-Tang, is widely used in China for the treatment of CAG. This standardized TCM formulation consists of *Pinelliae Rhizoma Praeparatum Cum Alumine* (Qing Banxia), *Scutellariae Radix* (Huangqin), *Coptidis Rhizoma* (Huanglian), *Zingiberis Rhizoma* (Ganjiang), *Curcumae Radix* (Yujin), and *Astragali Radix* (Huangqi), among other components. The formulation is developed based on long-term clinical practice and the core pathogenesis theory of “deficiency, stagnation, depression, and blood stasis” in TCM. It aims to strengthen the spleen and replenish deficiency, regulate gastrointestinal function, soothe the liver and relieve depression, promote the flow of qi, and resolve blood stasis to prevent disease progression. A multicenter randomized controlled trial confirmed that WW-1 significantly reverses gastric atrophy and IM, particularly in high-risk gastric corpus and incisura angularis lesions, with a favorable safety profile [12]. Experimental studies suggest that WW-1 may exert anti-inflammatory effects by inhibiting a disintegrin and metalloproteinase 17 activity and reducing tumor necrosis factor-α secretion [13]. In vitro studies also indicate that WW-1–containing serum can suppress S100A9 expression in M2 tumor-associated macrophages, thereby inhibiting proliferation and migration of GC cells and promoting apoptosis. However, robust clinical evidence supporting its efficacy and underlying mechanisms in patients with CAG, particularly after *H pylori* eradication, remains insufficient.

In addition, the digestive tract microbiota, including the gut, gastric, oral, and tongue-coating microbiota, has emerged as an important factor in the pathogenesis and progression of CAG. The gut microbiota functions as a complex and dynamic system that contributes to host metabolism, immune regulation, and homeostasis. Microbial metabolites, such as short-chain fatty acids and bile acids, play key roles in host-microbiota interactions [14,15]. Increasing evidence suggests that TCM formulations may exert therapeutic effects by modulating microbiota composition and metabolic activity [16]. Moreover, microbiota from different regions of the digestive tract, including saliva, tongue coating, and gastric mucosa, may also be involved in CAG progression [17-20].

Therefore, this study aims to evaluate the efficacy and safety of WW-1 in patients with CAG following successful *H pylori* eradication through a multicenter, randomized, double-blind, placebo-controlled trial. In addition, this study seeks to investigate the potential mechanisms of WW-1 by analyzing gastric mucosal DNA methylation and the composition of digestive tract microbiota. We hypothesize that WW-1 can improve histopathological outcomes in CAG and exert therapeutic effects by modulating epigenetic regulation and microbiota homeostasis.

## Methods

### Study Design

This trial adopted a multicenter, randomized, double-blind, placebo-controlled design with balanced allocation (1:1) across 2 arms, enrolling a total of 110 participants (55 per study group). This protocol is version 02, dated March 25, 2023. The study is registered with the Chinese Clinical Trial Registry (ChiCTR2400084357) on May 15, 2024. This protocol is described according to the SPIRIT (Standard Protocol Items: Recommendations for Interventional Trials) 2013 and SPIRIT-TCM (Standard Protocol Items for Clinical Trials with Traditional Chinese Medicine: Recommendations, Explanation and Elaboration) 2018 [21].

### Recruitment

**Figure 1:**
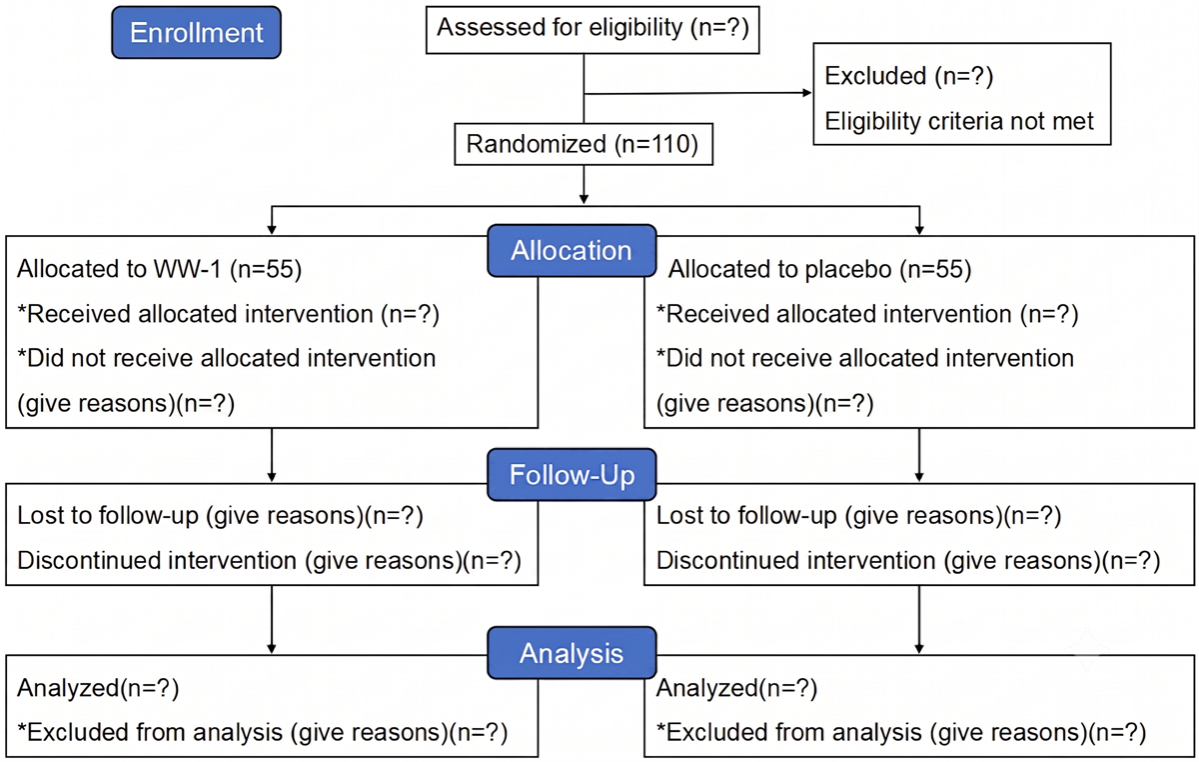
Participant flowchart. WW-1: Qi-Lian-Xiao-Pi prescription.

Recruitment is conducted through multiple channels, including public advertisements (posters, social media, and community outreach) and the identification of potential participants from gastroenterology outpatient and previous inpatient lists. Eligible patients are comprehensively informed about the study’s purpose, procedures, treatment duration, potential benefits, and risks. A gastroenterology specialist confirms the diagnosis for all patients who express interest. Written informed consent must be obtained from each participant prior to randomization. All patient information will be kept confidential. Patients may withdraw at any trial stage, and the reasons for patient withdrawal will be well documented. The flowchart of this trial is shown (Figure 1).

Participant enrollment is conducted within a multicenter framework involving 4 tertiary hospitals, each with an established gastroenterology service and access to a defined patient population with CAG. Recruitment is carried out through parallel approaches, including outpatient screening, inpatient record review, and community-based outreach. Screening and enrollment procedures are standardized across centers, with unified protocols applied at each site. Recruitment progress is monitored throughout the study period in relation to the planned timeline. As of the time of manuscript submission, patient enrollment is ongoing. The first patient was enrolled on April 20, 2025, and the study is projected to conclude in October 2026.

### Participants Selection

#### Diagnostic Criteria

##### Endoscopic Observation Grading Standard

Endoscopic atrophy was graded as mild, moderate, or severe according to the following criteria: mild: red-white appearance, predominantly white, visible vascular network, often focal. Moderate: red-white appearance, predominantly white, clearly visible vascular network, often diffuse, mucosal folds flattened/shallow. Type B atrophy extends from the antrum to the gastric angle level. Severe: above features plus granular/nodular hyperplastic changes. Type B atrophy extends to the upper/middle gastric body.

##### Pathological Diagnostic Standard (Centralized Blinded Assessment)

Pathological Diagnostic Standard evaluates 6 histological changes using a 4-point scale (0: none; +: mild, ++: moderate; +++: severe) via a visual analog scale.

##### Chronic Inflammation (Mononuclear Cell Infiltration)

Chronic inflammation was assessed according to the density and depth of mononuclear cell infiltration. Activity was evaluated based on neutrophil infiltration.

##### Atrophy (Reduction of Intrinsic Glands)

Atrophy was defined as the reduction of gastric intrinsic glands. Scores were classified as 0 (no reduction), + (reduction ≤1/3 of original glands), ++ (reduction >1/3 but ≤2/3 of original glands), and +++ (reduction >2/3 of original glands).

##### IM Grading

Scores were classified as 0 (absent), + (IM area ≤1/3 of gland/surface area), ++ (IM area >1/3 but ≤2/3), and +++ (IM area >2/3).

##### Dysplasia Grading

Dysplasia was graded as + (mild), ++ (moderate), and +++ (severe).

##### Endoscopic Biopsy Protocol (The Updated Sydney System)

Five biopsy specimens were obtained: 2 from antrum (3 cm from pylorus: 6 o'clock greater curve, 12 o'clock lesser curve), 2 from body (8 cm from cardia: 6 o'clock greater curve/mid-body; 4 cm proximal to angle: lesser curve), 1 from the angle. Additional biopsies from any visible lesions.

##### H Pylori Diagnosis

H pylori infection was considered positive based on either biopsy histology or special staining (eg, silver stain), 13C or 14C urea breath test, or a rapid urease test. Proton pump inhibitor, histamine H2 receptor antagonist, bismuth, and antibiotics had to be stopped at least 2 weeks before testing.

#### Participant Selection and Study Discontinuation Criteria

Eligibility criteria, participant withdrawal and dropout criteria, and conditions for trial suspension are summarized in Textboxes 1 and 2.

**Participant eligibility criteria.**

**Inclusion criteria**

- A history of H pylori infection with successful eradication confirmed at least 1 year prior to enrollment.
- Meet the above diagnostic criteria for chronic atrophic gastritis (CAG) at the time of eradication.
- Endoscopic and histopathological confirmation of gastric atrophy (reduction of intrinsic glands) with or without intestinal metaplasia, but without dysplasia, prior to enrollment.
- Age between 35 and 70 years, and gender is not limited.
- The participant is informed and voluntarily signs the informed consent form.
- Resident in the local area, able to ensure treatment follow-up, and possesses basic reading comprehension skills.
- Those who have not taken anticoagulants such as aspirin, warfarin, and those with no coagulation dysfunction.

**Exclusion criteria**

- Autoimmune gastritis (type A CAG).
- Patients with gastric and duodenal ulcers and upper gastrointestinal bleeding.
- Patients with high-grade intraepithelial neoplasia of the gastric mucosa or suspected malignant transformation by pathological diagnosis.
- Patients with serious primary diseases such as the heart, brain, liver, kidney, hematopoietic system, and respiratory system.
- Individuals presenting with comorbid conditions or psychiatric disorders that may compromise treatment assessment or study adherence, as determined by investigators, will be excluded.
- Female participants who are currently pregnant, breastfeeding, or planning conception within the study period will be excluded.
- Those with allergies or a history of allergies to multiple drugs (more than 2 or known drugs or ingredients in the study); Allergic to components of the product.
- Other circumstances that the investigator considers inappropriate for inclusion.
- Patients who are participating in clinical trials of other medicines.

**Participant discontinuation and trial suspension criteria.**

**Dropout criteria**

- Participants who demonstrate unwillingness or inability to continue trial participation, regardless of the underlying cause, are discontinued.
- Participants who discontinue therapeutic interventions and protocol-specified assessments without formal withdrawal notification are categorized as passive discontinuations.
- Cases that have been enrolled but have not completed the clinical protocol are considered to have dropped out if they have the above conditions. The specific reasons for participant dropout are rigorously documented.

**Voluntary withdrawal of participants**

- Participants become unwilling or unable to continue trial participation for any reason.
- Participants become lost to follow-up, meaning they discontinue treatment and study visits without formally withdrawing.

**Conditions for suspension**

- The occurrence of any serious adverse event during the treatment period.
- The identification of a major flaw in the clinical trial protocol that compromises the accurate evaluation of treatment efficacy.
- Any significant protocol deviation that occurs, if sustained, would compromise the validity of the treatment efficacy evaluation.
- The principal investigator deems it necessary to discontinue the study.
- A request to suspend the trial is issued by the drug regulatory authority.

### Sample Size Calculation

The superiority test of the outcome measure Operative Link on Gastritis Assessment (OLGA) improvement rate was used as a categorical variable, and the sample size of the control group was estimated by n1. n2 represents the sample size of the control group, k represents the ratio of the number of cases between the 2 groups (k=1), and the minimum clinically meaningful difference is set △=10%. According to the results of the team’s previous research, π1=45%, π2=10% [12]. By further incorporating a 2-sided significance level (α) of 0.05 and a statistical power of 80%, the calculation yielded n2=43 and n1=43. Considering the 20% clinical dropout rate, the sample size was determined to be 108 cases, that is, 54 cases in the experimental group and 54 cases in the control group.

For the superiority test of categorical variables with the improvement rate of the outcome measure Operative Link on Gastric Intestinal Metaplasia Assessment (OLGIM), in which △=10%, k=1, π1=42%, and π2=8% [12], using the same significance level (α=0.05) and statistical power (80%), the calculation yielded n2=44 and n1=44. Considering the 20% clinical dropout rate, the sample size was determined to be 110 cases, that is, 55 cases in the experimental group and 55 cases in the control group.

The larger calculated sample size was chosen to ensure adequate statistical power. Therefore, the sample size was 110 cases, 55 cases in the experimental group, and 55 cases in the control group.




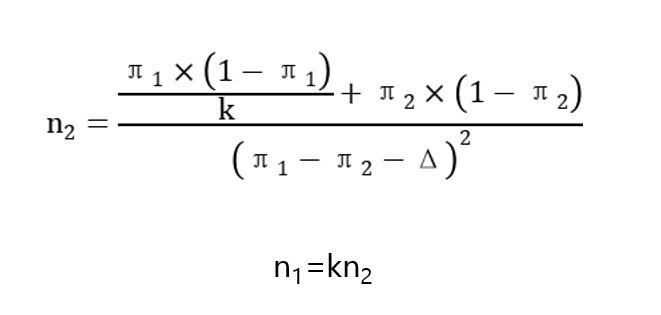




### Randomization, Allocation, and Blinding

In this study, case collection is being carried out in 4 institutions (Wangjing Hospital of China Academy of Chinese Medical Sciences, Shanxi Provincial Hospital of Traditional Chinese Medicine, Second Affiliated Hospital of Baotou Medical College, and Hubei Provincial Hospital of Traditional Chinese Medicine). The study uses a stratified block randomization method. Stratification is based on both the research center and the baseline OLGA/OLGIM stage. The third-party statistical agency used SAS software (SAS Institute Inc) to generate a random number list, and each clinical research center randomly allocates the qualified participants to the experimental group and the control group according to the ratio of 1:1. The trial is also designed as a double-blind trial. The blinding is conducted by independent personnel, and the blind record is formed. To ensure the integrity of the blinding, all medications and placebos are uniformly packaged. According to the Standard for the Use of Food Additives in the National Standard for Food Safety, the placebos are prepared from starch, dextrin, food coloring, bitters, etc, based on the type and volume of the raw materials. In appearance, color, dosage form, quality, taste, and odor, it is the same as or basically similar to the experimental drug, but does not contain the active ingredient of the experimental medicine.

All randomization code envelopes must remain strictly sealed, with any unblinding requiring full documentation of personnel, date/time, and rationale, immediately recorded in the case report form (CRF). Emergency unblinding is only permitted for life-threatening situations requiring knowledge of the investigational product, and must involve joint authorization by the clinician and study director, followed by participant discontinuation and detailed CRF documentation. Post study, all unsealed envelopes undergo verification and final blinded review by an independent audit team to ensure compliance with International Council for Harmonisation of Technical Requirements for Pharmaceuticals for Human Use-Good Clinical Practice and regulatory standards.

### Intervention Method

#### Overview

All participants receive standardized patient education and consultation regarding lifestyle, diet, and disease management throughout the study period.

On this basis, participants in the intervention arm receive active WW-1 granules, whereas the control arm receives visually identical placebo preparations. Both formulations were produced by Hunan Xinhui Pharmaceutical Co., Ltd. (Hunan, China) and authenticated using HPLC-MS analysis. The matched placebo replicates the active drug in all sensory characteristics (color, aroma, and flavor) and packaging. To maintain strict blinding integrity against the distinct sensory characteristics of the herbal formula, the placebo granules were formulated with a subtherapeutic dose (5%) of the WW-1, combined with matching pharmaceutical excipients. This 5% concentration aligns with the technical guidelines for TCM clinical trials issued by the National Medical Products Administration of China, providing necessary sensory fidelity without exerting any pharmacological therapeutic effect.

Both groups follow an identical administration regimen: oral intake of 1 sachet twice daily for 24 weeks. No additional pharmacological treatments for CAG are permitted during the trial. The overall timeline of this double-blind study, including treatment allocation and evaluation points, is presented in Table 1.

**Table 1:** Schedule of the multicenter, randomized, double-blind, placebo-controlled clinical trial.

Stage procedure	Screening period	Washout period	Treatment period						
	Visit 1 (–14 d ~ 0 d)	1 week	Visit 2 (0 day)	Visit 3 (4 weeks)	Visit 4 (8 weeks)	Visit 5 (12 weeks)	Visit 6 (16 weeks)	Visit 7 (20 weeks)	Visit 8 (24 weeks)
Screening for inclusion/exclusion	✔								
Sign informed consent form	✔								
Demographic data	✔								
Disease risk factors	✔								
Past medical history	✔								
Medical comorbidities and current medication	✔			✔	✔	✔	✔	✔	✔
C13/C14 urea breath test	✔								
Gastroscopy and pathology	✔								✔
Tongue coating, saliva, stomach and fecal microbiota collection	✔								✔
TCM^a^ syndrome scoring			✔	✔	✔	✔	✔	✔	✔
Vital signs	✔		✔	✔	✔	✔	✔	✔	✔
Blood routine	✔								✔
Urine routine	✔								✔
Stool routine and occult blood	✔								✔
Liver function tests (AST^b^ and ALT^c^)	✔								✔
Renal function tests (BUN^d^ and Cr^e^)	✔								✔
Electrocardiogram	✔								✔
Adverse events				✔	✔	✔	✔	✔	✔
Dispensing medication			✔	✔	✔	✔	✔	✔	
Combination therapy medication record			✔	✔	✔	✔	✔	✔	✔
Drug return and record				✔	✔	✔	✔	✔	✔
Trial summary									

^a^TCM: traditional Chinese medicine.

^b^AST: aspartate aminotransferase.

^c^ALT: Alanine aminotransferase.

^d^BUN: blood urea nitrogen.

^e^Cr: creatinine.

#### Drug Interaction Management Principles

During the trial, Chinese medicines and proprietary Chinese medicines with similar efficacy in the treatment of CAG or similar effects cannot be used. Gastric mucosal protectants and/or proton pump inhibitors can be used for a short period of time once a month for less than 7 days, and are recorded in detail on a CRF. If the participant has comorbid diseases and must continue to maintain these medications or treatments, the disease, the name of the medication, the dose used, the frequency of use, the route of administration, and the start and end times must be recorded in detail. Any changes in the participant’s medication throughout the study are also recorded in detail on the CRF for analysis and reporting at the time of summary. At each follow-up visit, the researchers keep a detailed record of whether the participants took their medications on time and count the number of medications returned. Keep a record of lost or unreturned medications. If the participant’s compliance is not good, the cause should be found and documented. The investigator should record the number of drugs issued and returned in the corresponding section of the CRF. Patient compliance is calculated as the percentage of the actual total medication taken relative to the total medication required by the study protocol, using the formula: (actual total medication taken/total protocol-required medication) × 100%.

### Outcome Evaluation

The primary outcome, OLGA improvement, is evaluated based on assessments at baseline (visit 1) and posttreatment (visit 8). The severity of gastric atrophy is assessed at baseline and post-treatment according to the OLGA staging system, with the overall stage at each time point determined by the most severe atrophic grade across all biopsy sites. Patients are categorized into three groups based on changes in OLGA stage: regression, defined as a reduction of ≥1 stage after treatment; stable, defined as no change in OLGA stage after treatment; and progression, defined as an increase of ≥1 stage after treatment.

Secondary outcomes include the improvement of gastric mucosal atrophy and IM (assessed at visits 1 and 8), as well as changes in TCM syndrome scores (assessed at visits 1, 2, 3, 4, 5, 6, 7, and 8). Based on the histopathological characteristics of CAG, a graded scoring system will be used for the pathological assessment. The severity of gastric mucosal atrophy and IM will be classified into 4 grades—absent, mild, moderate, and severe—which will be assigned scores of 0, 2, 4, and 6, respectively. In cases where multiple biopsy specimens or histological sections from a single patient exhibit varying degrees of lesions, the score corresponding to the most severe lesion will be assigned. The improvement in these pathological scores from baseline to posttreatment will be compared between the 2 groups. Additionally, clinical symptoms will be quantitatively evaluated using a standardized scoring method. The primary symptoms include stomach pain and fullness, while secondary symptoms encompass gastric discomfort, poor appetite, dry mouth, bitter taste in the mouth, belching, acid reflux, heartburn, and fatigue. The symptom grading criteria are formulated according to the *Guiding Principles for Clinical Research of New Traditional Chinese Medicines* (2002 edition). Specifically, primary symptoms will be comprehensively assessed based on their severity and frequency, and categorized into 4 grades (absent, mild, moderate, and severe), scoring 0, 2, 4, and 6 points, respectively. Secondary symptoms will be evaluated using the same 4-tier scale but will be scored as 0, 1, 2, and 3 points, respectively. The total symptom scores will be recorded at baseline and at 4-week intervals throughout the treatment period. Changes in the total symptom scores relative to baseline will be compared between the 2 groups to comprehensively assess symptom improvement.

### Safety Outcome

Vital signs examination, including temperature, respiration, heart rate, and blood pressure, is recorded at baseline and posttreatment (visits 1, 2, 3, 4, 5, 6, 7, and 8). The routine blood, urine, and stool tests, liver function tests (alanine aminotransferase and aspartate aminotransferase), and renal function tests (blood urea nitrogen and creatinine) are recorded at baseline (visit 1) and at the end of treatment (visit 8). Throughout the study, adverse events (AEs) will be continuously monitored and documented following treatment administration. Any participant exhibiting abnormalities post treatment—despite baseline normalcy—will undergo periodic assessments until values stabilize. All AEs will be actively tracked until complete resolution is achieved for each participant.

### Mechanism Investigation

To clarify the mechanism of WW-1 in treating patients with CAG, gastric mucosa, saliva, tongue coating, and stool are collected at baseline (visit 1) and at the end of the treatment (visit 8), followed by the Infinium MethylationEPIC BeadChip assay and metagenomic analysis. All sample collection, storage, and transportation processes are carried out in accordance with the standard operating procedure, which has been developed by the expert committee.

### Quality Control of Pathological Blind

Pathological histology is the gold standard for diagnosing CAG. However, consistency among pathologists in clinical practice is often inadequate, particularly in the assessment of atrophy. High-quality international studies related to CAG emphasize the necessity of multiple pathologists’ diagnoses. This study uses a centralized blind pathology approach, using a remote pathology consultation platform for the diagnosis and evaluation of CAG. Pathology experts from the pathology departments of Wangjing Hospital of the China Academy of Chinese Medical Sciences, the Cleveland Clinic in the United States, and Peking Union Medical College Hospital conduct blind diagnoses and reach a consensus. This process ensures the accuracy of pathological results before and after treatment, thereby enhancing the objectivity, rigor, and authenticity of efficacy assessments.

### Quality Control of the Trial

Prior to the trial, all study personnel received protocol-specific training. Investigators must accurately complete CRFs as unalterable primary records, with original laboratory/imaging reports attached. Clinically significant abnormal data require physician verification. Monitors conduct regular site visits, perform source data verification, and provide feedback through reports and meetings to ensure data quality.

To ensure data accuracy, investigators at all participating sites must complete the CRFs with complete honesty. Regular audits are conducted by monitoring personnel to verify the recorded information. When making corrections, the original entries must remain legible, with all modifications accompanied by the investigator’s signature and the date of amendment.

Once reviewed by the monitors, the CRFs are forwarded to the clinical trial data management team. The entire process—including communication between researchers, monitors, and data administrators—is meticulously documented. Prior to data entry, the administrators perform another round of validation. Any discrepancies or clarifications will be recorded as query forms and archived for future review. This structured approach ensures traceability and maintains the integrity of the clinical trial data throughout the study.

### Data Management

Within the drug clinical trial facilities of DHABUCM, the data management software EpiData (version 3.1; EpiData Association) is used, and a database is set up. Data entry personnel carry out independent double data entry. Once the data entry process is completed, a number of CRFs are sampled to assess the entry quality, and any existing issues are addressed. The database is reviewed by data administrators in collaboration with principal researchers. The original CRFs are filed and saved after data entry and verification as required. Electronic data files containing data banks, inspection procedures, analysis results, codebooks, and description files have several backups and are saved on different record media properly. All original files are saved according to “China’s Drug Clinical Trial Quality Management Standard” within the specified time.

### Statistical Analysis Plan

The approach of this study is to use an intention-to-treat analysis, ensuring that all participants are included in the analysis as originally assigned. Specialized statisticians will handle the statistical analysis in a blinded manner to maintain the integrity of the results. Regarding missing data, particularly for the primary histopathological end point, we will address this issue using the Last Observation Carried Forward method to impute missing data. This approach will help mitigate the potential bias introduced by incomplete data and ensure that the analysis remains robust. Using SAS software (version 6.12; SAS Institute Inc.), all statistical tests will be 2-sided, with a *P* value of ≤.05 considered to indicate statistical significance. Initially, we will assess the baseline balance to determine the comparability of the intervention and control groups in terms of their fundamental characteristics. For quantitative data, we will provide a comprehensive description including the count, mean, SD, median, and range (minimum-maximum), and use either a *t* test or a Wilcoxon rank sum test to compare the 2 groups. Qualitative data will be presented in terms of frequency and percentage, with a chi-square test or Fisher exact test used to evaluate differences between the groups. In the analysis of combined medication use, we will enumerate the number and percentage of occurrences and then apply a chi-square test or Fisher exact test to analyze any disparities between the groups. For the assessment of treatment compliance, we will enumerate the cases where the administered dosage of the experimental drug falls below 80%, exceeds 120%, or lies within the 80%-120% range and subsequently perform a chi-square test or Fisher exact test to identify any significant differences.

Regarding safety analysis, we will detail the incidence of AEs and serious AEs that occur during the treatment period. We will also present detailed tables outlining the specifics of AEs and serious AEs to provide a thorough understanding of the safety profile of the treatment.

For the high-dimensional and compositional microbiome data, we will adopt a genome-specific and interaction-focused analytical framework. Briefly, high-quality metagenome-assembled genomes will be reconstructed, and global shifts in the gut microbiota structure will be evaluated using permutational multivariate analysis of variance with permutations. Rather than relying solely on traditional differential abundance testing, coabundance networks will be constructed using Fastspar to account for complex microbial interactions. Subsequently, connected components clustering and weighted correlation network analysis will be applied to identify stably correlated genome clusters, or guilds [14].

### Ethical Considerations

This study adheres to the ethical standards described in the Declaration of Helsinki and relevant Chinese clinical research norms and regulations. The trial protocol has been formally reviewed and approved by the Ethics Committee of Wangjing Hospital, China Academy of Chinese Medical Sciences (approval WJEC-KT2024-022-P002, April 2, 2024). Written informed consent must be obtained from each participant prior to enrollment and randomization. Furthermore, strict measures are implemented to maintain the privacy and confidentiality of all research participants; personal identifying information is fully anonymized, and all participant data are securely stored and accessible only to authorized study personnel.

## Results

The funding began in November 2023. The study was officially initiated on April 20, 2025, with the enrollment of the first participant. This commencement followed the completion of essential preparatory measures, including the formulation of the study protocol, the registration of the clinical trial, the ethical review, and the establishment of the framework for participant recruitment. The study is projected to conclude in October 2026, following the completion of 24 weeks of treatment. The final study results, including efficacy outcomes, safety profiles, and mechanistic insights, are expected to be released after comprehensive data analysis and verification.

## Discussion

### Principal Findings

This study hypothesizes that the WW-1 will significantly improve the histopathological outcomes of CAG, specifically by reversing gastric mucosal atrophy and IM, in patients who have achieved successful *H pylori* eradication. Furthermore, we anticipate that the mechanistic evaluation will demonstrate that WW-1 exerts these therapeutic effects by modulating gastric mucosal DNA methylation profiles and restoring the homeostasis of the digestive tract microbiota.

### Comparison to Prior Work

Currently, the internationally recognized approach to intervening in the cancer transformation process of CAG primarily focuses on eradicating *H pylori*. However, research has demonstrated that, among individuals infected with *H pylori*, the number of patients with gastric atrophy continues to significantly increase even 1 year after eradication therapy [20]. In comparing changes in gastric mucosal lesions before and after eradication therapy, scholars often regard IM as a “point of no return” in the cascade model of GC. For instance, a meta-analysis involving 12 studies and 2658 patients revealed that eradicating *H pylori* significantly improved atrophy in the gastric body but did not alleviate atrophy or IM in the gastric antrum. A Japanese study found that gastric body IM, regardless of extent, was an independent predictor of GC development after *H pylori* eradication [22]. A long-term randomized controlled trial following 800 patients with CAG in a region of Colombia with high GC incidence for 20 years found that gastric mucosal lesion scores began to decline only 3 years after achieving *H pylori* negativity [6]. Therefore, delaying the inflammation-carcinoma transformation induced by *H pylori* represents a critical and challenging step in the prevention of GC. Actively and effectively conducting intervention studies targeting this transformation process is of great significance for GC prevention and control. Currently, there is a lack of specific therapeutic agents supported by high-quality evidence-based medicine. Existing treatment strategies are mainly focused on symptom relief and regular endoscopic and histopathological surveillance, with limited clinical efficacy. TCM, based on a holistic perspective, demonstrates multidimensional advantages in managing gastric precancerous lesions. Our previous multicenter trial, conducted in collaboration with Professor Barry Marshall’s team, indicated that a 24-week intervention with WW-1 significantly improved atrophy scores in the gastric body and angle compared to placebo [12]. Given its proven efficacy in reversing general gastric atrophy, we postulate that WW-1 holds substantial therapeutic potential for the treatment of CAG following *H pylori* eradication.

Crucially, the microbiota of multiple parts of the digestive tract plays an important role in the occurrence and development of CAG and precancerous lesions. The stomach constitutes a hostile environment for most microorganisms, as potent gastric acid efficiently eliminates indigenous microbes [17,18]. Consequently, traditional culture methods detect only a minimal microbial biomass in the highly acidic stomach. However, when gastric acidity is reduced, such as in cases of mucosal atrophy, the abundance of viable gastric microbiota increases substantially, accompanied by a shift in microbial composition [20]. Early studies on CAG and precancerous lesions focused on the microbiota of the stomach, but more recently, more research has begun to focus on the intestinal microbiota. Some studies have even shown that there is a stronger potential relationship between intestinal microbiota disorder and CAG and precancerous lesions than gastric microbiota. When the intestinal microbiota is out of balance, pathogenic bacteria can release a large number of cytotoxins, and related metabolites such as bile acids, polyamines, and specific enzymes can also promote CAG and precancerous lesions [23].

Moreover, the tongue coating microbiome could also serve as a potential noninvasive biomarker suitable for long-term monitoring of CAG and precancerous lesions. The findings of one study suggest that 21 tongue-coating microbiomes were differentially abundant by metagenomic sequencing between gastritis patients, including superficial gastritis, CAG, IM, and healthy individuals [19]. Notably, the study highlights *Campylobacter concisus* as a key microbial marker associated with the precancerous cascade of gastritis, a finding that was further validated through its detection in gastric fluid. The observed reduction in microbial diversity and the specific microbial correlations with symptoms such as bitter taste and dry mouth further emphasize the complexity and individuality of the microbiome’s role in gastritis. This discovery provides a scientific foundation for the TCM practice of tongue diagnosis. There is also obvious dysbiosis of the oral microbiota of CAG and other precancerous lesions patients, which might involve the pathogenesis of CAG and other precancerous lesions. Sung et al [20] have demonstrated that following the eradication of *H pylori*, a distinct cluster of oral bacteria within the gastric mucosa is associated with the development and persistence of CAG and IM, including *Peptostreptococcus, Streptococcus, Parvimonas, Prevotella, Rothia,* and *Granulicatella*. From the above, it is apparent that microbial communities throughout the entire gastrointestinal tract can exert a substantial influence on the pathogenesis of CAG and precancerous lesions.

The important mechanism of action of TCM formulas in treating diseases, especially digestive diseases, is through intervention in the digestive tract microbiota. The constituents of TCM formulas modulate the composition and structure of gastrointestinal microbiota, thereby influencing the distant functions of affected organs and tissues through the systemic actions mediated by gastrointestinal microbiota. Following oral administration, TCM formulas interact with gastrointestinal microbiota in several ways: (1) TCM can modulate the composition of gastrointestinal microbiota, (2) TCM can influence the metabolic activities of gastrointestinal microbiota, and (3) gastrointestinal microbiota is capable of transforming compounds found in TCM formulas [24]. In this study, we focus on the regulatory effects of WW-1 on 2 core microbiota groups—keystone functional microbiota and pathogenic functional microbiota. Research suggests that these 2 microbiota groups interact with each other, forming a “seesaw” model that influences human health [14]. WW-1 may help restore the dynamic balance of the microbiota by modulating these core groups, thereby improving both the digestive system and overall health.

Concurrently, DNA methylation plays a critical role in the initiation and progression of various tumors. Consequently, modulating DNA methylation has emerged as a promising therapeutic strategy for oncology. Accumulating evidence suggests that TCM mediates potent antitumor effects through the regulation of DNA methylation processes [25]. TCM concepts of balance and holism are consistent with the equilibrium of DNA methylation modifications within the tumor microenvironment. TCM has been demonstrated to represent a class of potential and reliable agents for the modulation of epigenetic alterations. Therefore, one of the objectives of this study is to use DNA methylation microarray technology to investigate whether the TCM formula WW-1 can regulate the DNA methylation status or levels of specific key gene loci or genes (eg, tumor suppressor genes) in human gastric mucosal tissues, thereby elucidating the underlying mechanisms of WW-1 in preventing and controlling the inflammation-carcinoma transformation process.

The digestive tract microbiota has a significant influence on epigenetic modifications, including DNA methylation in the host, which plays a key role in the development of multiple diseases. Intestinal microbiota can modulate gut homeostasis and inflammation by regulating DNA methylation [26]. Our study will provide novel insights into the potential correlation between digestive tract microbiota and DNA methylation and elucidate the specific regulatory mechanisms underlying CAG and precancerous lesions for the first time.

The collection of digestive tract microbiota strictly adheres to established protocols. Our team possesses extensive research expertise in the field of microbiota, ensuring the implementation of standardized procedures and establishing a solid foundation for obtaining high-quality data [14,27]. Simultaneously, our standardized gastric mucosal sample collection and rigorous double-blind pathological evaluation of the gastric mucosa are pivotal for assessing WW-1 efficacy and lay the foundation for further elucidating the mechanism of WW-1. Accurate and adequate gastric mucosal biopsies are essential for determining the extent of gastric mucosal lesions and for risk stratification of GC using the OLGA and OLGIM staging systems. According to the updated Sydney system gastritis classification method [28], our study takes 5 biopsies from the gastroscope, including 2 from the antrum, 2 from the body, and 1 from the angle of the stomach, so that the efficacy of TCM can be observed overall and in different parts. The mucosa of the antrum minor, the site selected for the collection of the gastric flora under endoscopy, also meets the collection standards certified by high-quality research [29].

A multicenter, randomized, double-blind, placebo-controlled trial is used, with central pathological assessment by blinding as the primary outcome indicator of pathological histological improvement. This study aims to obtain evidence-based clinical data regarding the efficacy and safety of WW-1 for patients with CAG following *H pylori* eradication, and to reveal its mechanism of action in the prevention of GC at the level of DNA methylation and the digestive tract microbiota. Following completion of the trial’s statistical analysis, we will communicate results to participants, health care professionals, and the public through peer-reviewed publications. If successful, the findings of this clinical trial will be valuable in the treatment of CAG and drug development, providing new ideas for the diagnosis and treatment of CAG and the prevention of GC.

### Strengths and Limitations

This study focuses on CAG after *H pylori* eradication to evaluate the therapeutic potential of TCM intervention, offering new strategies for early GC prevention. In previous studies, early screening and prevention of GC have mainly concentrated on pathological indicators and clinical manifestations. However, this study adopts a more comprehensive research approach by evaluating multiple dimensions, including clinical symptoms, molecular mechanisms, and microbiome, offering a more holistic perspective.

A major strength of this trial is the use of DNA methylation profiling to elucidate the mechanisms underlying TCM-mediated GC risk reduction. We hypothesize that TCM modulates the methylation status of key genes (eg, tumor suppressor genes and oncogenes) in the gastric mucosa, thereby regulating their expression and potentially halting disease progression. Furthermore, moving beyond standard pathology and isolated biomarkers, we have established a comprehensive digestive tract microbiota assessment framework. Integrating data from the tongue coating, saliva, gastric, and intestinal microbiota provides a more complete picture of how TCM impacts the gastrointestinal microenvironment, laying the groundwork for future mechanistic research.

However, several limitations should be noted. First, despite the multicenter design, the sample size remains relatively small. Larger-scale cohorts are needed to validate the generalizability of our findings across diverse populations. Second, while we explore the impact of TCM on DNA methylation, the specific interactions between these epigenetic modifications and microbiota alterations require further investigation.

### Future Directions

Future research should focus on the following areas: first, further validation of the therapeutic effects of WW-1 on precancerous gastric lesions in larger clinical studies. Second, future studies should consider incorporating long-term follow-up to assess the safety and effectiveness of TCM intervention with prolonged use. Finally, therapeutic strategies targeting specific methylation sites or microbiota imbalances could become a research hotspot in TCM intervention and warrant further exploration.

### Dissemination Plan

Following the completion of the trial and comprehensive statistical analysis, the primary findings regarding the clinical efficacy and safety of WW-1, alongside the mechanistic insights, will be submitted for publication in high-impact, peer-reviewed scientific journals. We also plan to present our key findings at major international academic conferences, such as Digestive Disease Week, to share our methodology and results with the global gastroenterology community.

## Abbreviations

AE: adverse event
BUN: blood urea nitrogen
CAG: chronic atrophic gastritis
CRF: case report form
GC: gastric cancer
IM: intestinal metaplasia
OLGA: Operative Link on Gastritis Assessment
OLGIM: Operative Link on Gastric Intestinal Metaplasia Assessment
SPIRIT: Standard Protocol Items: Recommendations for Interventional Trials
SPIRIT-TCM: Standard Protocol Items for Clinical Trials with Traditional Chinese Medicine: Recommendations, Explanation and Elaboration
TCM: traditional Chinese medicine
WW-1: Qi-Lian-Xiao-Pi prescription

## Data Availability

Data sharing is not applicable to this article, as no datasets were generated or analyzed for this article.

## References

[1] Gullo I, Grillo F, Mastracci L, Vanoli A, Carneiro F, Saragoni L, Limarzi F, Ferro J, Parente P, Fassan M (2020). Precancerous lesions of the stomach, gastric cancer and hereditary gastric cancer syndromes. Pathologica.

[2] Bray F, Laversanne M, Sung H, Ferlay J, Siegel RL, Soerjomataram I, Jemal A (2024). Global cancer statistics 2022: GLOBOCAN estimates of incidence and mortality worldwide for 36 cancers in 185 countries. CA Cancer J Clin.

[3] Yin Y, Liang H, Wei N, Zheng Z (2022). Prevalence of chronic atrophic gastritis worldwide from 2010 to 2020: an updated systematic review and meta-analysis. Ann Palliat Med.

[4] Yang H, Yang WJ, Hu B (2022). Gastric epithelial histology and precancerous conditions. World J Gastrointest Oncol.

[5] Shah SC, Piazuelo MB, Kuipers EJ, Li D (2021). AGA clinical practice update on the diagnosis and management of atrophic gastritis: expert review. Gastroenterology.

[6] Piazuelo MB, Bravo LE, Mera RM, Camargo MC, Bravo JC, Delgado AG, Washington MK, Rosero A, Garcia LS, Realpe JL, Cifuentes SP, Morgan DR, Peek RM, Correa P, Wilson KT (2021). The Colombian chemoprevention trial: 20-Year follow-up of a cohort of patients with gastric precancerous lesions. Gastroenterology.

[7] Sugano K, Suzuki C, Ota M, Iwakiri R (2025). Gastric cancer risk after Helicobacter pylori eradication in gastritis and peptic ulcer: a retrospective cohort study in Japan. BMC Gastroenterol.

[8] Correa P (1992). Human gastric carcinogenesis: a multistep and multifactorial process-first American Cancer Society award lecture on cancer epidemiology and prevention. Cancer Res.

[9] Yang L, Ying X, Liu S, Lyu G, Xu Z, Zhang X, Li H, Li Q, Wang N, Ji J (2020). Gastric cancer: epidemiology, risk factors and prevention strategies. Chin J Cancer Res.

[10] Liu Y, Huang T, Wang L, Wang Y, Liu Y, Bai J, Wen X, Li Y, Long K, Zhang H (2025). Traditional Chinese medicine in the treatment of chronic atrophic gastritis, precancerous lesions and gastric cancer. J Ethnopharmacol.

[11] Guo J, Chen X, Wu M, Wang D, Zhao Y, Li Q, Tang G, Che F, Xia Z, Liang Z, Shi L, Jiang Q, Chen Y, Liu X, Ren X, Ouyang M, Wang B, You S, Billot L, Wang X, Liu Z, Jing H, Meng W, Tian S, Liu E, Xiang Y, Tang X, Xie T, Cui W, Zheng Y, Cao J, Zhang J, Wen Z, Huang T, Wang L, You C, Pan S, Cai Y, Lu Y, Hankey GJ, Al-Shahi Salman R, Anderson CS, Song L, CHAIN investigators (2024). Traditional Chinese medicine FYTF-919 (Zhongfeng Xingnao oral prescription) for the treatment of acute intracerebral haemorrhage: a multicentre, randomised, placebo-controlled, double-blind, clinical trial. Lancet.

[12] Yang Y, Qin Z, Du S, Fang S, Ma Y, Liu Z, Zhou W, Liu X, Tay ACY, Tang Y, Wang C, Su J, Liu F, Shu J, Tian X, Yang Q, Liu H, Liu L, Yu T, Li Z, Zhang Z, Li Y, Wang X, Xiong G, Liu J, Duan DD, Zhang P, Wei W (2026). Efficacy of Chinese medicine WW-1 in managing gastric atrophy and intestinal metaplasia in patients with chronic atrophic gastritis: a multicenter, randomized, double-blind, placebo-controlled trial. Pharmacol Res.

[13] Du S, Wang T, Li Z, Li T, Miao Z, Chen Y, Zhu S, Wei W, Deng H (2025). Therapeutic potential of qilianxiaopi formula: targeting ADAM17-mediated chronic inflammation in atrophic gastritis. Pharmaceuticals (Basel).

[14] Wu G, Xu T, Zhao N, Lam YY, Ding X, Wei D, Fan J, Shi Y, Li X, Li M, Ji S, Wang X, Fu H, Zhang F, Shi Y, Zhang C, Peng Y, Zhao L (2024). A core microbiome signature as an indicator of health. Cell.

[15] Nicolas GR, Chang PV (2019). Deciphering the chemical lexicon of host-gut microbiota interactions. Trends Pharmacol Sci.

[16] Li X, Wu D, Niu J, Sun Y, Wang Q, Yang B, Kuang H (2021). Intestinal flora: a pivotal role in investigation of traditional Chinese medicine. Am J Chin Med.

[17] Tsuda A, Suda W, Morita H, Takanashi K, Takagi A, Koga Y, Hattori M (2015). Influence of proton-pump inhibitors on the luminal microbiota in the gastrointestinal tract. Clin Transl Gastroenterol.

[18] Koga Y (2022). Microbiota in the stomach and application of probiotics to gastroduodenal diseases. World J Gastroenterol.

[19] Cui J, Cui H, Yang M, Du S, Li J, Li Y, Liu L, Zhang X, Li S (2019). Tongue coating microbiome as a potential biomarker for gastritis including precancerous cascade. Protein Cell.

[20] Sung JJY, Coker OO, Chu E, Szeto CH, Luk STY, Lau HCH, Yu J (2020). Gastric microbes associated with gastric inflammation, atrophy and intestinal metaplasia 1 year after eradication. Gut.

[21] Dai L, Cheng C, Tian R, Zhong LL, Li Y, Lyu A, Chan A, Shang H, Bian Z (2019). Standard protocol items for clinical trials with traditional Chinese medicine 2018: recommendations, explanation and elaboration (SPIRIT-TCM Extension 2018). Chin J Integr Med.

[22] Shichijo S, Hirata Y, Niikura R, Hayakawa Y, Yamada A, Ushiku T, Fukayama M, Koike K (2016). Histologic intestinal metaplasia and endoscopic atrophy are predictors of gastric cancer development after helicobacter pylori eradication. Gastrointest Endosc.

[23] Jia ET, Liu ZY, Pan M, Lu JF, Ge QY (2019). Regulation of bile acid metabolism-related signaling pathways by gut microbiota in diseases. J Zhejiang Univ Sci B.

[24] Feng W, Ao H, Peng C, Yan D (2019). Gut microbiota, a new frontier to understand traditional Chinese medicines. Pharmacol Res.

[25] Zhu D, Li A, Lv Y, Fan Q (2022). Traditional Chinese medicine: a class of potentially reliable epigenetic drugs. Front Pharmacol.

[26] Ansari I, Raddatz G, Gutekunst J, Ridnik M, Cohen D, Abu-Remaileh M, Tuganbaev T, Shapiro H, Pikarsky E, Elinav E, Lyko F, Bergman Y (2020). The microbiota programs DNA methylation to control intestinal homeostasis and inflammation. Nat Microbiol.

[27] Zhao L, Zhang F, Ding X, Wu G, Lam YY, Wang X, Fu H, Xue X, Lu C, Ma J, Yu L, Xu C, Ren Z, Xu Y, Xu S, Shen H, Zhu X, Shi Y, Shen Q, Dong W, Liu R, Ling Y, Zeng Y, Wang X, Zhang Q, Wang J, Wang L, Wu Y, Zeng B, Wei H, Zhang M, Peng Y, Zhang C (2018). Gut bacteria selectively promoted by dietary fibers alleviate type 2 diabetes. Science.

[28] Dixon MF, Genta RM, Yardley JH, Correa P (1996). Classification and grading of gastritis. The updated Sydney System. International Workshop on the Histopathology of Gastritis, Houston 1994. Am J Surg Pathol.

[29] Guo Y, Zhang Y, Gerhard M, Gao J, Mejias-Luque R, Zhang L, Vieth M, Ma J, Bajbouj M, Suchanek S, Liu W, Ulm K, Quante M, Li Z, Zhou T, Schmid R, Classen M, Li W, You W, Pan K (2020). Effect of on gastrointestinal microbiota: a population-based study in Linqu, a high-risk area of gastric cancer. Gut.

